# A novel integrative procedure for identifying and integrating three-dimensions of objectively measured free-living sedentary behaviour

**DOI:** 10.1186/s12889-017-4994-0

**Published:** 2017-12-28

**Authors:** Anna Myers, Catherine Gibbons, Edward Butler, Michelle Dalton, Nicola Buckland, John Blundell, Graham Finlayson

**Affiliations:** 1 Centre for Sport and Exercise Science, Faculty of Health and Wellbeing, Sheffield Hallam University, Collegiate Hall S10 2BP, Sheffield, UK; 2 Appetite Control and Energy Balance Research, School of Psychology, Faculty of Medicine and Health, University of Leeds, Leeds, UK; 3 Endava Ltd, London, UK; 4 School of Social and Health Sciences, Leeds Trinity University, Leeds, UK; 5 Department of Psychology, Faculty of Science, University of Sheffield, Sheffield, UK

**Keywords:** Free-living sedentary behaviour, Sitting, Measurement, Methodology, Integration, activPAL, SenseWear

## Abstract

**Background:**

The widely accepted definition of sedentary behaviour [SB] refers to any waking behaviour characterized by an energy expenditure ≤1.5 metabolic equivalents [METs] while in a sitting or reclining posture. At present, there is no single field-based device which objectively measures sleep, posture and activity intensity simultaneously. The aim of this study was to develop a novel integrative procedure [INT] to combine information from two validated activity monitors on sleep, activity intensity and posture, the three key dimensions of SB.

**Methods:**

Participants in this analysis were initially recruited from a series of three studies conducted between December 2014 and June 2016 at the University of Leeds. Sixty-three female participants aged 37.1 (13.6) years with a body mass index of 29.6 (4.7) kg/m^2^ were continuously monitored for 5–7 days with the SenseWear Armband [SWA] (sleep and activity intensity) and the activPAL [AP] (posture). Data from both activity monitors were analysed separately and integrated resulting in three measures of sedentary time. Differences in Sedentary time between the three measurement methods were assessed as well as how well the three measures correlated.

**Results:**

The three measures of sedentary time were positively correlated, with the weakest relationship between SED^SWA^ (awake and <1.5 METs) and SED^AP^ (awake and sitting/lying posture) [r(61) = .37,*p* = .003], followed by SED^SWA^ and SED^INT^ (awake, <1.5 METs and sitting/lying posture) [r(61) = .58,*p* < .001], and the strongest relationship was between SED^AP^ and SED^INT^ [r(61) = .91,p < .001]. There was a significant difference between the three measures of sedentary time [*F*(1.18,73.15) = 104.70,p < .001]. Post-hoc tests revealed all three methods differed significantly from each other [p < .001]. SED^SWA^ resulted in the most sedentary time 11.74 (1.60) hours/day, followed by SED^AP^ 10.16 (1.75) hours/day, and SED^INT^ 9.10 (1.67) hours/day. Weekday and weekend day sedentary time did not differ for any of the measurement methods [*p* = .04–.25].

**Conclusion:**

Information from two validated activity monitors was combined to obtain an objective measure of free-living SB based on posture and activity intensity during waking hours. The amount of sedentary time accumulated varied according to the definition of SB and its measurement. The novel data integration and processing procedures presented in this paper represents an opportunity to investigate whether different components of SB are differentially related to health end points.

## What are the new findings?

- A procedure has been developed to integrate information on three dimensions of free-living sedentary behaviour (wakefulness, posture and low activity intensity) using two validated activity monitors.
- Amount of sedentary time differs according to the measurement method.
- This procedure operationalizes the current leading definitions of sedentary behaviour for use in research.

## Impact on clinical practice

- Future research will be able to clarify which dimensions of sedentary behaviour are detrimental to health; low activity intensity (and therefore energy expenditure) or sitting.

## Background

Sedentary behaviour (SB) is highly prevalent in the twenty-first century accounting for between 46% - 72% of the waking day [1–3]. The prevalence of SB is of particular concern as there is evidence to suggest SB is an independent risk factor for deleterious health outcomes including metabolic syndrome, all-cause mortality, cardiovascular disease, type 2 diabetes and obesity [4–6]. The Sedentary Behaviour Research Network (SBRN), an organization of researchers and health professionals, define SB as “any waking behaviour characterized by an energy expenditure ≤1.5 metabolic equivalents (METs) while in a sitting or reclining posture” [7]. Sedentary time is defined as “the time spent for any duration (e.g., minutes per day) or in any context (e.g., at school or work) in sedentary behaviours” [8].

Free-living SB is notoriously difficult to quantify due to the complexity of human movement. Previous studies have used TV viewing as a proxy measure to reflect total sedentary time [9, 10] however, TV viewing is not representative of overall sedentary time and it is also associated with other health related behaviours such as increased energy intake, particularly from fat [11, 12]. To address the limitations of self-report proxy measures of SB, objective measurement methods are increasingly being used [2, 13, 14]. Devices used to measure free-living movement behaviour reflect different facets of SB and can provide quite different estimates of sedentary time [15]. The SenseWear Armband (SWA) uses information from a triaxial accelerometer (as well as other physiological information) to estimate sleep time and activity intensity and defines SB as activities with an intensity of <1.5 METs (see [16–18]). Alternatively, the activPAL (AP) uses a triaxial accelerometer to measure posture and defines SB based on a sitting or reclining posture (see [19–21]). Separately, each activity monitor has been shown to accurately measure the specific dimension of SB they specialise in, however, they are not able to capture multiple components of SB [20, 22, 23]. The multi-component definition of SB makes accurate assessment of sedentary time complex because different components of SB do not always covary; it is possible to be seated whilst expending >1.5 METs and it is also possible to stand whilst expending <1.5 METs [24, 25]. In other words, it is possible to be inactive whilst not in a seated posture; and alternatively to show some activity (>1.5 METs) whilst actually being seated. This issue, reflected by the concepts of passive standing and active sitting, is incorporated into the very recently published terminology consensus from the SBRN [8]. Therefore, there is a need for measurement tools that provide information on activity intensity and posture simultaneously as well as indicating whether the wearer is awake or asleep.

Surprisingly, there is no single objective measurement device that accurately measures sleep, posture (sitting/reclining) and activity intensity (<1.5 METs) simultaneously. Recent research has demonstrated combining information from multiple devices yielded a more accurate measure of SB and the authors encouraged further research using the “multi-method” approach [26]. Furthermore, data from the ActiGraph and AP have been shown to have greater accuracy when classifying activity intensity and estimating energy expenditure (EE) when data from both monitors were integrated [27]. However, these innovative data integration methods were suited to laboratory based research and not free-living conditions in which activities can be measured over multiple days. There is a need to develop a measurement method that can provide information on waking hours as well as posture and activity intensity under free-living conditions to better understand how SB, as defined by the SBRN, impacts on health outcomes.

The aim of this study was to develop a novel integrative procedure to combine data from the SWA and AP to identify and quantify SB based on both posture and activity intensity during waking hours.

## Methods

### Participants

Participants who were included in this analysis were initially recruited from a series of three studies conducted between December 2014 and June 2016 by the Appetite Control and Energy Balance Research team at the University of Leeds. General recruitment strategies included emails circulated on University mailing lists and poster advertisements. General inclusion criteria were: women, aged between 18 and 70 years, body mass index (BMI) between 18.5 and 45.0 kg/m^2^, premenopausal status, reporting good health, no contraindications to exercise and not taking medication known to effect metabolism or appetite. In the present analysis, we used each study’s baseline data from participants who had ≥5 days (including ≥1 weekend day) of valid SWA and AP data. All participants provided their written informed consent and all studies were approved by either the School of Psychology (University of Leeds) or NHS (NRES Yorkshire and the Humber) Ethics Committees (14–0099, 14–0090 and 09/H1307/7).

### Study design

The three studies included in this cross-sectional study followed the same systematic protocol according to standardised operating procedures. Participants attended the research unit twice over the course of one week. Free-living SB was measured continuously for a minimum of 5 days for >22 h/day with the SWA and AP simultaneously.

On the morning of day one, participants were provided with a physical activity diary and fitted with a SenseWear Armband mini (SWA; BodyMedia, Inc., Pittsburgh, PA) and activPal micro (AP; PAL Technologies Ltd., Glasgow, UK) and instructed to continue their normal daily living activities during the measurement period. Participants returned to the lab on day 7 or 8 to return the activity monitors and completed PA diary.

### Activity monitors

Participants wore the SWA on the posterior surface of their upper non-dominant arm for a minimum of 22 h per day for ≥6 days (except for the time spent showering, bathing or swimming). For the SWA data to be valid >22 h of data per day had to be recorded on at least five days (midnight to midnight) including at least one weekend day. The SWA measures motion (triaxial accelerometer), galvanic skin response, skin temperature and heat flux. Proprietary algorithms available in the accompanying software (SenseWear Professional 8.0, algorithm v5.2) calculate EE and classify the intensity of activity. SB was classified as time spent in activities <1.5 METs excluding sleep [28, 29]. The SWA has been shown to perform better than accelerometer-only activity monitors when classifying activity into minutes of SB, light, moderate and vigorous PA [22]. The SWA only records data when it is in contact with the skin and therefore provides a direct measure of compliance.

The AP was placed in a nitrile sleeve and attached to the midline anterior aspect of the upper thigh on the non-dominant leg with a hypafix waterproof dressing. Participants were instructed to wear the AP at all times. If they removed the device they were asked to record the day, time and reason for removing in the activity diary provided. The AP is a small (45 × 25 × 5 mm), light (7.7 g), thigh-mounted triaxial accelerometer which directly measures the postural element of SB. Accelerometer-derived information about thigh position and acceleration are used to determine body posture (sitting or lying (it is unable to distinguish between sitting and lying), standing and stepping), transitions between different postures, and number of steps using proprietary algorithms within the accompanying software (activPAL software version 7.2.32, Intelligent Activity Classification). Compliance with the AP wear protocol was determined by cross-checking any prolonged periods of sitting/lying (>2 h) with SWA data from the same period. If the SWA recorded movement (i.e. stepping) and activity intensity was >1.5 METs during this period it would indicate the AP had been removed and that days data was removed. The AP has excellent correlation (R^2^ = .94) and agreement (underestimated sitting by only 2.8%) with direct observation for sitting/lying time, upright time, sitting/lying to upright transitions and for detecting reductions in sitting [19, 20, 30].

### Data handling

**Fig. 1:**
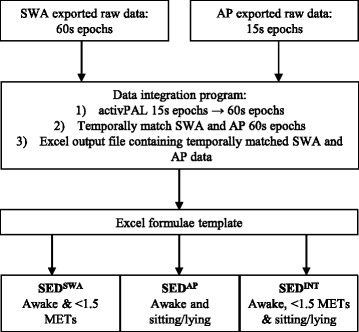
Represents the data integration and processing procedure developed to combine information from the SWA and AP to produce three SB variables, all of which excluded sleep: i) SED^SWA^ < 1.5 METs, ii) SED^AP^ sitting/lying, iii) SED^INT^ < 1.5 METs and sitting/lying

Data from the SWA and AP were combined to create an integrated SB variable (SED^INT^) which classified behaviour as sedentary when a 60 s epoch from the SWA registered activity <1.5 METs whilst awake and the AP registered activity in a sitting or lying posture. This data integration and processing technique is represented in fig. 1 and explained in more detail below.

#### Data preparation

Both activity monitors were set up on the same computer such that their internal time stamps would match to facilitate data processing. Data from both activity monitors was exported from the proprietary software by day into .xls (SWA) and .csv (AP) format. Each participant had 5–7 pairs of exported files from the SWA and AP, each pair representing one day of raw data (24 h from midnight to midnight). To enable the temporal matching of data from the SWA and AP, the AP data had to be condensed from 15 s epochs to 60 s epochs. A program (graphical user interface; GUI) was developed that summed the time spent sitting, standing, stepping and number of steps for every four 15 s epochs to create 60 s epochs and then temporally matched the newly created 60 s AP epochs with the SWA 60 s epochs.

#### Data integration

A set of algorithms to perform the data merge operations as well as a simple GUI were developed. Both components were written using the Python language and delivered as a self-contained executable file. The data merging operation comprised of a set of algorithms that executed a number of tasks. Firstly, using Python libraries ‘xlrd’ and ‘csv’, each source document (.xls or .csv file) was converted into ordered arrays (or dictionaries). Each row of sensor data was represented as a key value pair; the key being the timestamp, the value being the sensor outcome variables. Every four 15 s epoch from the AP data were summed resulting in a new summary row designated for zero seconds past the minute (or ‘hh:mm:00’). The key representing this summary was then searched for in the 60 s epoch array and when matched the associated values (or rows) were appended. Finally, the combined array of key value pairs was then written to an Excel file using the Python library ‘xlwt’. Any headers or footers for the source data were retained and copied back into the output file upon completion of the merge operation.

To execute the merging operation a spreadsheet was created for each participant detailing the source data file names and locations. This spreadsheet was then parsed as the input file for the merge operation. The resulting output file contained 5–7 days of free-living SB data from the SWA and AP. Each tab within the file contained data for one 24 h period in 60 s epochs for both the SWA and AP.

#### Data processing

A Microsoft Excel template containing formulae was created to calculate average sedentary time per day based on three different criteria. The first required SWA data only (SED^SWA^; minutes/day) and was calculated by summing the number of minutes per day categorised as awake and <1.5 METs. The second required the sleep variable from the SWA and posture information from the AP (SED^AP^; minutes/day) and was calculated by summing the number of minutes per day categorised as awake and sitting/lying. The full 60 s had to be sitting/lying to be counted as a sedentary epoch. The third variable required information on sleep and activity intensity (<1.5 METs) from the SWA and posture (sitting/lying) from the AP (SED^INT^; minutes/day) and was calculated by summing the number of minutes per day categorised as awake, <1.5 METs and sitting/lying. Average minutes of SB per day for each SB variable were then calculated by summing total sedentary time for each day and dividing by the number of days the activity monitors had been worn. The Microsoft Excel template also contained formulae to determine how SB was accumulated based on pre-determined bout lengths (1–5, 6–10, 11–20, 21–40 and >40 min) and provided information on the frequency of bouts and the number of minutes accumulated in a given bout category.

### Statistical analysis

Data are reported as mean (SD) throughout. Statistical analysis was performed using IBM SPSS for Windows (Chicago, Illinois, Version 21). For reasons of scientific rigour and to reduce the likelihood of false positives, we only regarded relationships as meaningful with a *p* value < .01. Characteristics of the study population were summarised using descriptive statistics. Differences in SED^SWA^, SED^AP^ and SED^INT^ methods were examined using repeated measures ANOVA with Bonferroni post-hoc tests. Paired sample t-tests were performed to identify differences in sedentary time accumulated on weekdays compared with weekend days. Pearson correlations were performed to examine the associations between the different measures of sedentary time. The difference in sedentary time measured using the SWA alone and AP alone for each participant are presented to identify whether either measurement method systematically differed from the other. Additionally, Bland-Altman plots were reported to provide information on the systematic bias and limits of agreement between SED^SWA^, SED^AP^ and SED^INT^ measures of sedentary time.

## Results

**Fig. 2:**
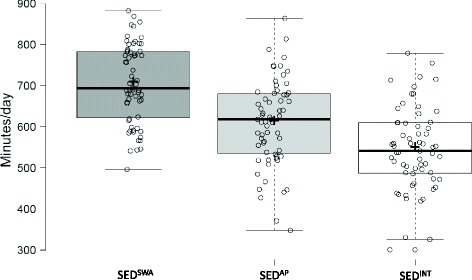
Difference in the amount of sedentary time when measured using the SED^SWA^, SED^AP^ and SED^INT^ methods. All measures were significantly different from each other [*p* < .01]. Centre lines show the medians; box limits indicate the 25th and 75th percentiles as determined by R software; whiskers extend 1.5 times the interquartile range from the 25th and 75th percentiles, outliers are represented by dots; crosses represent sample means; data points are plotted as open circles. *n* = 63 sample points

Sixty-three female participants, aged 37.1 (13.6) years with a BMI of 29.6 (4.7) kg/m^2^, had ≥5 days (including at least one weekend day) of valid SWA and AP data. Average wear time of the SWA was 23.6 (0.3) hours/day (98.4% of possible wear time) and average wear period was 6.5 (0.7) days. Participants were sedentary (excluding sleep) for an average of 11.7 (1.6) hours/day (70.7% of waking hours), 10.2 (1.8) hours/day (61.2% of waking hours) and 9.1 (1.7) hours/day (54.8% of waking day) determined by the SED^SWA^, SED^AP^ and SED^INT^ methods, respectively (see figure 2).

The associations between measures of free-living SB are displayed in Table 1. All three measures of sedentary time were significantly positively correlated. The weakest association was between SED^SWA^ and SED^AP^ [*p* = .003], followed by SED^SWA^ and SED^INT^ [*p* < .001] and the strongest association was between SED^AP^ and SED^INT^ [*p* < .001].

**Table 1:** Correlation between different measures of free-living sedentary time

	SED^SWA^ (min/d)	SED^AP^ (min/d)	SED^INT^ (min/d)
SED^SWA^ (min/d)	–	.37*	.58**
		[.13, .56]	[.39, .72]
SED^AP^ (min/d)	–	–	.91**
			[.86, .95]

*N* = 63; Data are Pearson correlation (r); 95% confidence intervals. **p < .001; *p < .01. SWA, SenseWear Armband; AP, activPAL; INT, integrated data

There was a significant difference between average daily sedentary time determined by the different measurement methods [*F*(1.18, 73.15) = 104.70, *p* < .001]. Post-hoc tests using the Bonferroni correction revealed all three methods were significantly different from each other [p < .001]. SED^SWA^ recorded the most sedentary time, followed by SED^AP^, and the least amount of sedentary time was recorded using the SED^INT^ method (see figure 2). The three different methods also produced significantly different measures of sedentary time from each other when weekdays [F(1.19, 73.52) = 91.67, p < .001] and weekend days [F(1.24, 76.74) = 100.75, p < .001] were analysed separately. Post-hoc tests using the Bonferroni correction revealed all three methods produced significantly different measures of sedentary time on weekdays and weekend days [p < .001]. SED^SWA^ recorded the most sedentary time, followed by SED^AP^, and the least amount of sedentary time was recorded using the SED^INT^ method during weekdays and weekend days. Paired sample t-tests revealed the amount of sedentary time accumulated on weekdays compared with weekend days did not differ significantly when measured using any of the measurement methods [*p* = .04–.25].

**Fig. 3:**
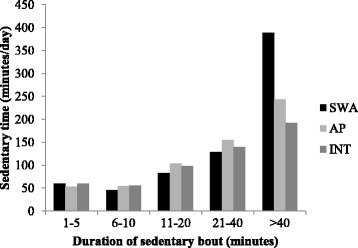
Sedentary time accumulated in different bout categories when measured using the SED^SWA^, SED^AP^ and SED^INT^ methods

Sedentary time accumulation measured using the three measurement methods is displayed in fig. 3. As the duration of the sedentary bout categories increases so too did the amount of sedentary time accumulated in that category. A similar amount of time was accumulated in the shorter sedentary periods for all three methods. SED^SWA^ gave the highest measure of total sedentary time and this is reflected in the longest sedentary bout category with SED^SWA^ registering more time in the >40 min category compared with SED^AP^ an SED^INT^. When posture is included in the sedentary time measurement method (SED^AP^ and SED^INT^) less sedentary time is accumulated in the longest bout category.

**Fig. 4:**
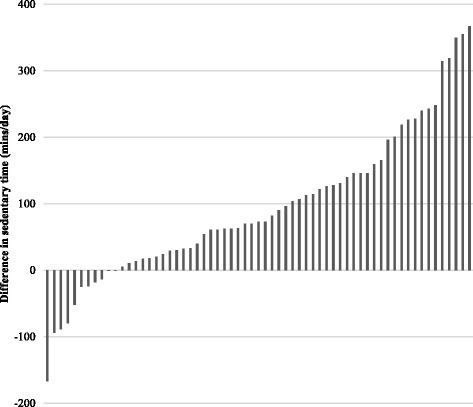
Individual differences in sedentary time when determined by the SED^SWA^ and SED^AP^ methods. Each grey bar represents 1 participant and the difference in sedentary time was calculated by subtracting SED^AP^ from SED^SWA^

**Fig. 5:**
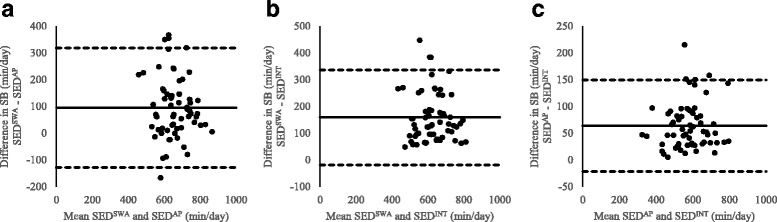
Bland-Altman plots of the difference in sedentary time when measured using the A) SED^SWA^ and SED^AP^ method, B) SED^SWA^ and SED^INT^ method and C) SED^AP^ and SED^INT^ method against the mean of the two measures being compared. The solid black line represents the mean difference (bias) and the upper and lower dashed black line represent the upper and lower 95% limits of agreement (LOA)

The difference in SED^SWA^ and SED^AP^ determined sedentary time were calculated for each participant to identify whether classification of SB based on either activity intensity or posture alone (during waking hours) resulted in systematically different estimates of sedentary time for the same measurement period. Figure 4 displays the individual variation in the difference in sedentary time estimated based on activity intensity (SWA) or posture (AP). The figure shows that 11 of the participants accumulated more sedentary time when it was measured based on posture, whereas 52 accumulated more sedentary time when it was measured based on activity intensity. Figure 5 displays a series of Bland-Altman plots showing the degree of systematic bias and limits of agreement between all measures of sedentary time. The largest average difference (bias) in measures of sedentary time was between SED^SWA^ and SED^INT^ (158.7 min/day) and the smallest difference in sedentary time was between SED^AP^ and SED^INT^ (63.8 min/day). Panel B and C of fig. 5 show that SED^SWA^ and SED^AP^ consistently provide higher estimates of sedentary time when compared with SED^INT^. However, panel A shows that in some individuals SED^AP^ provides a greater estimate of sedentary time and in others SED^SWA^ is greater.

## Discussion

The primary aim of the current study was to develop a method to integrate information from two validated activity monitors on both posture and activity intensity during waking hours, the three key dimensions of SB as defined by the SBRN [7].This study demonstrates it is possible to identify free-living sedentary time during waking hours based on posture and activity intensity alone, and in combination using two validated activity monitors and a novel integrative procedure. At present, there is no single field-based measurement device that accurately captures both the activity intensity and postural element of SB during waking hours. The development of a device that can capture these dimensions of SB has been highlighted as a research priority [31]. Until such a device is available, integrating information from multiple activity monitors, such as the method presented in the current paper, may overcome SB measurement limitations. Recently, Ellingson et al. combined postural information from the AP with information on activity intensity from the ActiGraph and found that estimates of activity intensity during sedentary and light activities were more accurate than estimations based on ActiGraph data alone [27]. Although this research demonstrates a methodological advancement in the measurement of SB, its utility in free-living conditions is unknown. The integrative procedure presented in the current paper represents a feasible method for all three dimensions of SB to be measured under free-living conditions.

According to the SBRN SB refers to “any waking behaviour characterized by an energy expenditure ≤1.5 METs while in a sitting or reclining posture” [7]. Although this definition has been widely accepted, other definitions are still being used, for example, Pate et al. define it as “activities that involve energy expenditure at the level of 1.0-1.5 METs” [29]. Whilst both definitions include low activity intensity, there is still debate whether SB should encompass activities at ≤1.5 METs whilst standing [31]. The recently published terminology consensus from the SBRN acknowledges that standing can be either active (>2 METs) or passive (<2 METs), however, it remains unclear whether passive standing should be classified as an active or sedentary behaviour (particularly when the intensity is <1.5 METs) [8]. The method described in the current paper allowed us to investigate the extent to which estimates of SB differed based on the definition and measurement technique used. There was a significant difference between average daily sedentary time determined by the different measurement methods. SED^SWA^ recorded the most sedentary time, followed by the SED^AP^, and the least amount of sedentary time was recorded by the SED^INT^ method. Furthermore, more sedentary time was accumulated in prolonged bouts when determined by the SED^SWA^ method. The volume of SB in the current study was large, but not dissimilar to other studies and sedentary time did not differ between weekdays and weekend days [16, 32, 33]. Participants were sedentary for between 9.1 h/day (54.8% of waking hours) and 11.7 h/day (70.7% of waking hours) depending on the measurement criteria. It is understandable that SED^SWA^ reflects a larger amount of SB since it would include instances of standing (as well as sitting) but with a MET of <1.5. The difference in sedentary time when estimated by different measurement methods carries important implications for the association between SB and health outcomes. Literature reporting the relationship between SB and health outcomes may arrive at different conclusions depending on the measurement device being used and therefore the component of SB being measured. Indeed, previous research has identified differences in associations between SB and cardio metabolic risk when measuring sedentary time subjectively and objectively [34]. However, differences in associations between health outcomes and different measures of objectively determined sedentary time have not been examined. Research to determine the specific properties of SB which relate to diminished health is a key priority [31, 35]. This will inform researcher’s decisions on the most appropriate device to use for their specific research question. Furthermore, existing recommendations to reduce sitting [36] could be updated and refined to provide more specific information about what activity should displace sitting to benefit health.

A strength of the current study is that it shows clearly that the amount of sedentary time identified varies with the particular device used; in turn, this has implications for associations with health and disease endpoints. A limitation of this study is that the sample is all female university employees and therefore, the results of this study may only apply to a similar demographic. Furthermore, the epochs were collapsed from 15 s to 60 s for the AP data but we do not feel that this resulted in a significant change in the degree of resolution.

## Conclusion

In this study, we have demonstrated a procedure to obtain a measure of free-living SB based on both activity intensity and posture during waking hours by integrating data from two validated activity monitors. This platform is flexible and can be expanded as new tracking technologies become available. Measures of sedentary time using different objective measurement techniques are not measuring the same components of SB. Indeed, the three measures of SB in the current study differed significantly. Determining whether the postural element of SB contributes to negative health outcomes attributed to SB or whether sitting is a marker for low EE remains a research priority. The novel data integration and processing procedures presented in this study represents an opportunity to investigate whether different components of SB are more strongly related to health outcomes than others.

## Abbreviations

AP: activPAL
BMI: body mass index
EE: energy expenditure
GUI: graphical user interface
METs: metabolic equivalents
SB: sedentary behaviour
SED^AP^: sedentary time measured using the activPAL
SED^INT^: sedentary time measured using the integrated data
SED^SWA^: sedentary time measured using the SenseWear armband
SWA: SenseWear armband
